# The impact of continuously‐variable dose rate VMAT on beam stability, MLC positioning, and overall plan dosimetry

**DOI:** 10.1120/jacmp.v13i6.4023

**Published:** 2012-11-06

**Authors:** Christopher Boylan, Alan McWilliam, Emily Johnstone, Carl Rowbottom

**Affiliations:** ^1^ The Christie NHS Foundation Trust Manchester and Manchester Academic Health Science Centre (MAHSC) Faculty of Medical and Human Sciences University of Manchester Manchester Manchester UK

**Keywords:** volumetric‐modulated arc therapy, dose rate, intensity‐modulated radiotherapy, treatment planning, dosimetric verification

## Abstract

A recent control system update for Elekta linear accelerators includes the ability to deliver volumetric‐modulated arc therapy (VMAT) with continuously variable dose rate (CVDR), rather than a number of fixed binned dose rates (BDR). The capacity to select from a larger range of dose rates allows the linac to maintain higher gantry speeds, resulting in faster, smoother deliveries. The purpose of this study is to investigate two components of CVDR delivery — the increase in average dose rate and gantry speed, and a determination of their effects on beam stability, MLC positioning, and overall plan dosimetry. Initially, ten VMAT plans (5 prostate, 5 head and neck) were delivered to a ${\text{Delta}}^{4}$ dosimetric phantom using both the BDR and CVDR systems. The plans were found to be dosimetrically robust using both delivery methods, although CVDR was observed to give higher gamma pass rates at the 2%/2 mm gamma level for prostates (p < 0.01). For the dual arc head‐and‐neck plans, CVDR delivery resulted in improved pass rates at all gamma levels (2%/2 mm to 4%/4 mm) for individual arc verifications (p < 0.01), but gave similar results to BDR when both arcs were combined. To investigate the impact of increased gantry speed on MLC positioning, a dynamic leaf‐tracking tool was developed using the electronic portal imaging device (EPID). Comparing the detected MLC positions to those expected from the plan, CVDR was observed to result in a larger mean error compared to BDR (0.13 cm and 0.06 cm, respectively, p < 0.01). The EPID images were also used to monitor beam stability during delivery. It was found that the CVDR deliveries had a lower standard deviation of the gun‐target (GT) and transverse (AB) profiles (p < 0.01). This study has determined that CVDR may offer a dosimetric advantage for VMAT plans. While the higher gantry speed of CVDR appears to increase deviations in MLC positioning, the relative effect on dosimetry is lower than the positive impact of a flatter and more stable beam profile.

PACS numbers: 87.56.bd; 87.55.km; 87.55.Qr

## I. INTRODUCTION

Dynamic arc radiotherapy has undergone several significant advancements since it was first proposed in 1995.^(1)^ Many of the developments have related to formalizing and improving the efficiency of inverse planning,^2^, ^3^ such that highly modulated and conformal dose distributions can now be achieved for a range of sites.^4^, ^6^ Just as significant are the advances in linear accelerator design — particularly in the ability of linac control systems to now reliably vary gantry speed, dose rate, and aperture shape simultaneously over the treatment arc.^(7)^ With the ability to deliver complex dose distributions efficiently and with a significant reduction in treatment time, arc radiotherapy is allowing many departments to improve their provision of intensity‐modulated radiotherapy.^(8)^

One of the commercial solutions for arc radiotherapy is Elekta VMAT. Previously, the Elekta VMAT solution only allowed the linac to select from fixed dose rate bins during delivery.^(9)^ The selection of dose rate bin and gantry speed for each control point is determined by the required change in multileaf collimator (MLC) shape and the number of monitor units to deliver. The binned dose rate (BDR) system, which is a feature of the Elekta Desktop 7.01 software, allows the dose rate to be reduced by factors of 2, such that for a maximum linac dose rate of $600\;\text{MU}.\min^{‐1}$, the available bins are $600\;\text{MU}.\min^{‐1}$, $300\;\text{MU}.\min^{‐1}$, $150\;\text{MU}.\min^{‐1}$, $75\;\text{MU}.\min^{‐1}$, and $37\;\text{MU}.\min^{‐1}$. A number of studies have shown good dosimetric results with BDR VMAT, using a variety of measurement techniques.^10^, ^12^

A more recent version of VMAT, packaged with the Integrity linac control software, allows for a much larger range of dose rates to be selected. Rather than five fixed dose rate bins, Integrity allows 255 bins to be selected from a nominal range of $37\;\text{MU}.\min^{‐1}$ to $600\;\text{MU}.\min^{‐1}$. The initial, and most prominent, impact of continuously variable dose rates (CVDR) is the much reduced treatment times. This is due to the linac being able to switch between smaller dose rate intervals, and thus maintain a higher gantry speed during treatment. A recent report by Bertelsen et al.^(13)^ has shown that CVDR VMAT provides good dosimetry and faster, smoother deliveries when applied to a number of clinical plans.

There is evidence to suggest that a higher average dose rate, which CVDR provides, can provide better beam stability during VMAT delivery. In particular, Bedford and Warrington^(14)^ reported that beam symmetry was poorer in the low dose rate bins for the BDR system. Generally, VMAT delivery preferentially selects higher dose rates, as this is closer to the conditions at linac calibration (i.e., $600\;\text{MU}.\min^{‐1}$). Significant deviations from these calibration conditions, as Bedford and Warrington show, may lead to increased beam asymmetry and, hence, poorer dosimetry. It has been suggested that the increase in average dose rate offered by CVDR may therefore provide a dosimetric advantage.^(13)^

Conversely, an increase in average dose rate leads to an increase in gantry speed, and concern has been expressed that this may adversely affect the dynamic positioning of MLCs over treatment.^13^, ^15^, ^16^ A recent study by Pasler et al.^(15)^ saw an improvement in dosimetry for VMAT prostate plans as delivery time was reduced (i.e., average dose rate and gantry speed was increased), but complex head‐and‐neck plans did not benefit from faster delivery. For these patients, dosimetry was poorer when delivered with a higher dose rate. This was attributed to some MLCs not reaching their intended position at each control point. An increase in MLC positioning errors with the move to higher gantry speeds was also reported by Bertelsen et al.^(13)^ for the Elekta Integrity system, and has also been observed with faster deliveries on the Varian RapidArc system.^(16)^ With the trend towards faster VMAT treatments, the dosimetric impact of these MLC errors warrants further investigation.

The purpose of this study is to investigate the impact of CVDR on beam stability and MLC positioning accuracy, when compared to the BDR system. Initially, dosimetric verification was carried out on ten VMAT plans. In order to more fully understand the effects of increased dose rate and increased gantry speed, further tests were carried out utilizing the linac's electronic portal imaging device (EPID). Dynamic leaf positioning accuracy was investigated by tracking the MLCs over the course of delivery. Using the same EPID acquisitions, the effect of increased dose rate on beam stability was also characterized over the ten patient plans. The relative impact of each of these parameters on dosimetric performance could then be assessed.

## II. MATERIALS AND METHODS

Ten patient plans were randomly selected, which consisted of five previously treated prostate VMAT patients, and five head‐and‐neck patients who had previously been treated with IMRT but were replanned with VMAT as part of a planning study. The plans were generated using Pinnacle version 9.0, utilizing the SmartArc optimization module (Philips Medical Systems, Madison, USA). The prostate patients were planned using a single arc technique, gantry rotating from 182° to 178°, with 4° between each control point, and a collimator angle of 45°. The beam energy was 10 MV and the final dose calculation was made using the adaptive collapsed cone convolution algorithm. A prescription of 57 Gy in 19 fractions to the prostate was set, with further dose levels covering the seminal vesicles, per group 3 of the CHHIP trial protocol.^(17)^ The mean number of monitor units (and standard deviation) for the prostate patients was 465.1±25.5  MU.

The five head‐and‐neck plans all involved complex shapes requiring a higher degree of modulation. All were three dose levels and consisted of three hypopharynx, one oropharynx, and one supraglottis. These were planned with a two arc solution, with gantry rotation from 182° to 178° and a collimator angle of 10° in both arcs. The control point spacing was again 4°, and the beam energy was 6 MV. 66 Gy was prescribed to PTV1, 60 Gy to PTV2, and 54 Gy to PTV3 using a simultaneous integrated boost (SIB) technique, in 30 fractions. On average, the total monitor units were 529.2±66.2  MU for the head‐and‐neck plans.

The ten plans were delivered on an Elekta Synergy linear accelerator (Elekta, Crawley, UK) which was fitted with a MLCi head (1 cm leaf thickness). The linac had recently been upgraded to the Integrity control software such that in ‘Service Mode’, it was possible to deliver plans with either BDR or CVDR. Delivery times and dose rates were recorded for each plan.

### A. Verification

Dosimetric verification was performed using each delivery method on the ${\text{Delta}}^{4}$ verification phantom (Scandidos, Uppsala, Sweden). The Delta^4^ phantom consists of two planes of silicon diodes in a cylindrical PMMA phantom. With the application of appropriate correction factors, a pseudo three‐dimensional analysis can be performed against the planned dose, and a gamma value calculated. This device has previously been shown to be an effective method for VMAT dosimetric verification.^(18)^ The Delta^4^ was set up at the isocenter of the linac and an inclinometer was fixed to the head to monitor gantry angle. Within the Delta^4^ software, a correction factor was applied based on the linac's recorded output for that day. No automatic alignment of the measured dataset was performed. Gamma analysis was performed at the 2%/2 mm, 3%/3 mm, and 4%/4 mm levels for each of the plans, with measurement points <20% of the maximum dose excluded from analysis.

### B. EPID MLC tracking

**Figure 1:**
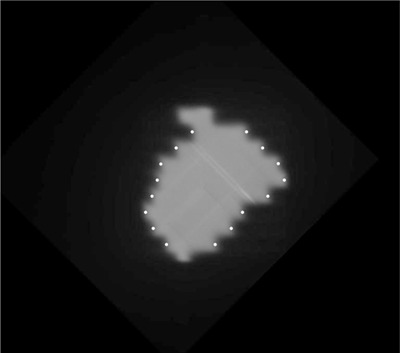
A single portal image acquired during VMAT delivery, with the MLC positions identified (white dots).

A software tool has previously been developed and validated at this center to determine MLC positions using the EPID.^19^, ^20^ For this study, the software has been expanded to allow for tracking of MLC positioning during VMAT delivery. EPIDs have been shown to provide a sensitive and independent means of determining MLC positioning *in vivo* during radiotherapy.^21^, ^22^ With the Elekta iView system, a movie was acquired over the course of each VMAT delivery with a frame recorded approximately every 0.47 s. For each frame of the movie (Fig. 1) a histogram of pixel intensities was taken such that the exposed area could be identified. The field edge, and therefore the MLC positions, was then determined by thresholding the image at 50% of the modal pixel intensity.

As the iView system does not record the linac gantry angle for each image, the gantry angle was determined by using the Service Graphing function within the linac control system. Service Graphing records the state of various linac parameters every 0.25 s during treatment, so it was possible to ‘tag’ each EPID image with the appropriate gantry angle. The VMAT plans were retrieved from a commercial record and verify system (MOSAIQ), and interrogated to determine the expected position of the MLCs during treatment. As the plan file only contains MLC data at each control point (i.e., every 4°), it was necessary to interpolate the MLC positions for images acquired between these gantry angles.

The accuracy of the EPID at determining MLC positions was found to be within 0.5 mm compared to film measurements, and reproducibility of measurements was < 0.01 mm.^(19)^ Prior to use with the VMAT plans, the system was tested under dynamic conditions using both a conformal (10×10 cm) and dynamic arc (a 2 cm sliding window defined by MLCs, similar to that described in Bedford and Warrington^(14)^). For the conformal arc, mean MLC deviation was determined to be ‐0.04 mm with a standard deviation (st. dev.) of 0.3 mm. For the sliding window the mean MLC deviation was ‐0.1 mm with a st. dev. of 1.2 mm.

### C. Beam flatness and stability

**Figure 2:**
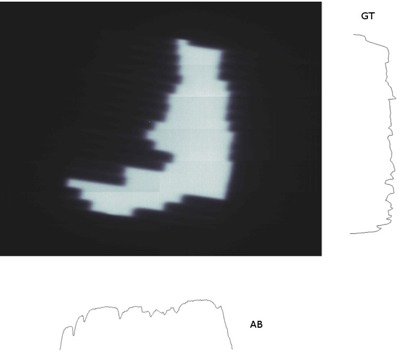
Flatness monitoring of a portal image. The pixel intensity is integrated over the whole exposed field area in the GT and AB directions to determine the beam profile. The standard deviation of each profile is then calculated to measure the flatness.

Using the same data from the portal imager, beam stability was assessed over each treatment arc for the binned dose rate deliveries and the continuously variable dose rate. Software was written which analyzes each frame from the EPID movie and monitors the profile of the beam in the gun‐target (GT) and transverse (AB) directions. Again, a histogram of the signal intensity in the image was used to identify the exposed area of the field, so that the effects of the penumbra and noise outside the field could be excluded. Then, the image was integrated across all rows (for the GT profile) and all columns (for the AB profile), taking a mean signal intensity per exposed pixel (Fig. 2). The standard deviation of each of these 1D profiles was recorded, and the process was then repeated over all of the frames of the EPID movie. The fluctuation of the beam profile could then be compared between the BDR and CVDR deliveries.

Due to the small sample size, the results of the gamma analysis, MLC deviations, and beam stability were statistically compared between BDR and CVDR over all deliveries using a non‐parametric Wilcoxon signed‐rank test. Significance was taken as p < 0.05. Where applicable, the standard deviation of results has been quoted in parentheses.

## III. RESULTS

**Figure 3:**
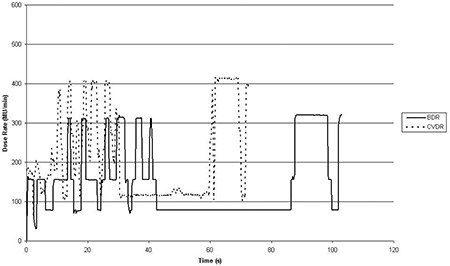
Dose rate varying with time for the BDR and CVDR deliveries of one of the head‐and‐neck arcs.

Delivery times using continuously variable dose rate were reduced compared to the binned dose rate system (Table 1). The mean reduction in delivery time for both the prostate plans and for the head‐and‐neck plans was 30.2%. Figure 3 shows how the dose rate varies over one of the head‐and‐neck deliveries. As expected, the CVDR deliveries have smaller steps between dose rate bins and a higher average dose rate. The mean dose rate for the CVDR deliveries was 266 $\pm 67\;\text{MU}.\min^{‐1}$ compared to 192 $\pm 55\;\text{MU}.\min^{‐1}$ for the BDR deliveries (p < 0.01 over all patients). Both delivery techniques were capable of switching between dose rate bins in less than 0.25 s (i.e., below the resolution of the Service Graphing function).

**Table 1:** Delivery times for the VMAT plans delivered with BDR and CVDR.

*Plan*	*Time (s)*	
	*Binned Dose Rate*	*Continuously Variable Dose Rate*
Prostate 1	118.9	80.8
Prostate 2	119.9	86.8
Prostate 3	119.6	83.2
Prostate 4	115.9	87.6
Prostate 5	122.6	78.4
Mean	119.4	83.4
Head and Neck 1	201.0	141.5
Head and Neck 2	210.3	146.0
Head and Neck 3	205.8	141.8
Head and Neck 4	204.5	144.3
Head and Neck 5	205.0	143.0
Mean	205.3	143.3

Results from the Delta^4^ verifications are shown in Table 2. For the prostate patients, no statistically significant difference was observed at the 3%/3 mm gamma analysis between the BDR and CVDR deliveries. At 2%/2 mm, there was an improvement in gamma pass in favor of CVDR (2.0% pixels failing versus 5.2%, p < 0.01). For head‐and‐neck plans, a statistically significant improvement in gamma pass was observed with CVDR at all gamma levels for individual arc verifications on the Delta^4^. However, the combined dose distributions (summing the contributions from both arcs) did not reflect this difference. The Delta^4^ verifications were found to be reproducible, with intercomparison of repeat deliveries giving gamma pass rates of 100% at 2%/2 mm.

**Table 2:** ${\text{Delta}}^{4}$ verification results for all plans delivered with BDR and CVDR. Values shown are the mean percent measurement points failing gamma analysis (± 1 st. dev.).

*Prostate*			*BDR*			*CVDR*			*p*
2%/2 mm			5.2±2.5%			2.0±1.5%			<0.01
3%/3 mm			0.6±0.8%			0.0±0.0%			>0.2
*Head and Neck*	*Arc 1*			*Arc 2*			*Combined*		
	*BDR*	*CVDR*	*p*	*BDR*	*CVDR*	*p*	*BDR*	*CVDR*	*p*
2%/2 mm	24.0±10.3%	23.4±6.0%	<0.01	17.2±10.5%	15.5±6.4%	<0.01	6.7±2.1%	6.5±2.6%	>0.2
3%/3 mm	7.0±5.1%	6.5±1.8%	<0.01	3.9±5.3%	3.1±1.7%	<0.01	1.1±0.4%	1.0±0.6%	>0.2
4%/4 mm	1.7±1.6%	1.4±0.5%	<0.01	1.0±1.3%	0.8±0.5%	<0.01	0.2±0.1%	0.1±0.1%	>0.2

The mean MLC positioning deviations are shown in Table 3. Over all deliveries, CVDR deliveries resulted in larger mean MLC deviations than BDR deliveries (p < 0.01). For the head‐and‐neck plans, this difference was more pronounced than for the prostates, with a mean deviation of 0.06 cm measured for the BDR system versus 0.13 cm for the CVDR system. Averaged over all patients, the st. dev. of MLC positioning errors was similar for both BDR and CVDR (p < 0.2).

**Table 3:** Mean and st. dev. of leaf positioning errors as determined by the EPID MLC tracking.

*Prostate Plan*	*BDR*		*CVDR*	
	*Mean Positional Error (cm)*	*St. Dev. (cm)*	*Mean Positional Error (cm)*	*St. Dev. (cm)*
1	0.08	0.18	0.09	0.17
2	0.07	0.24	0.08	0.21
3	0.09	0.17	0.09	0.22
4	0.07	0.76	0.09	0.31
5	0.08	0.21	0.09	0.17
Mean	0.08	0.31	0.09	0.22
*Head and Neck Plan*				
1	0.12	0.71	0.17	0.97
2	0.02	0.22	0.10	0.33
3	0.04	0.27	0.13	0.33
4	0.05	0.26	0.11	0.21
5	0.06	0.29	0.13	0.34
Mean	0.06	0.35	0.13	0.44

**Figure 4:**
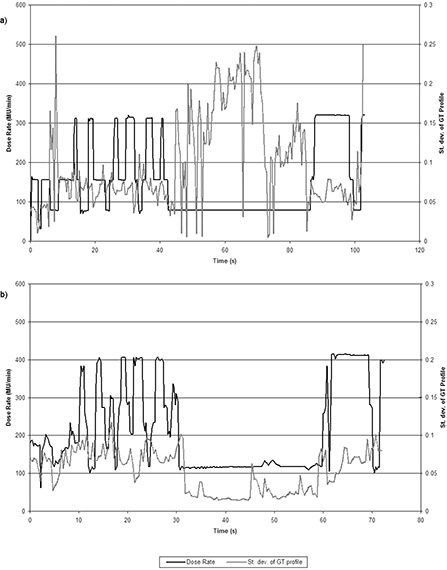
Standard deviation of the GT profiles over a head‐and‐neck arc, plotted alongside dose rate for the BDR delivery (a), and for the CVDR delivery (b).

**Figure 5:**
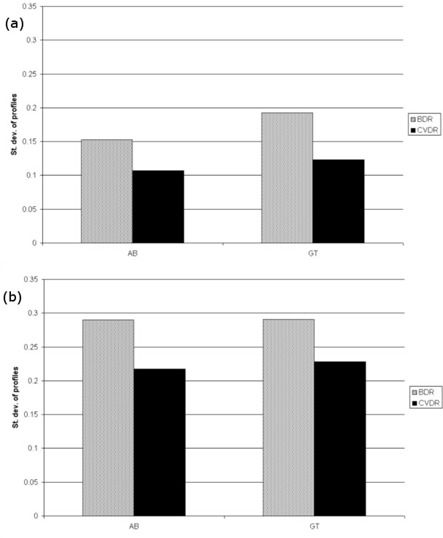
Mean of the standard deviation of the AB and GT profiles for (a) all prostate patients, and (b) all head‐and‐neck patients.

Using the portal images acquired during delivery, the standard deviation of the GT and AB profiles was calculated over each treatment arc. Figure 4 shows how the standard deviation varies over one of the head‐and‐neck arcs, for both CVDR and BDR. Over all the deliveries, the mean and maximum st. dev. of the profiles was higher for BDR deliveries compared to the CVDR deliveries (p < 0.05 for both prostate and head‐and‐neck deliveries). In all cases, st. dev. was larger in the GT profiles than in the AB direction (Fig. 5).

## IV. DISCUSSION

As expected, a reduction in delivery time was observed using continuously variable dose rate (~ 30.2%), which is in agreement with previous predicted and measured results.^13^, ^23^ This reduction is due to the ability to select a larger range of dose rates. On average, the increase in mean dose rate was 38.6% using CVDR compared to BDR.

**Figure 6:**
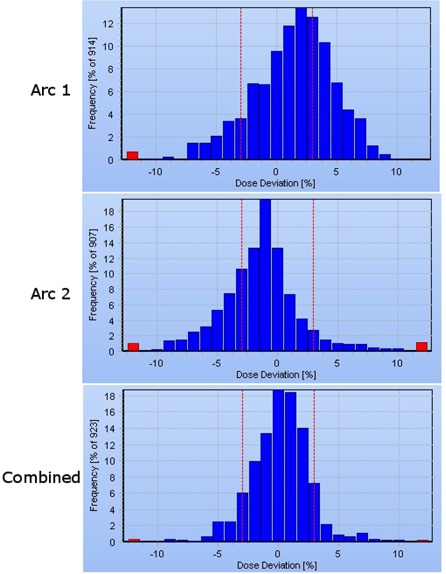
Delta^4^‐ measured dose deviations from one head‐and‐neck plan. The two individual arcs measure systematically low and high, such that the combined dose deviation is acceptable.

In general, dosimetric verification was found to be satisfactory for both the BDR and CVDR systems. Following this center's requirements for gamma evaluation, all prostate and head‐and‐neck deliveries were considered clinically acceptable using the Delta^4^ phantom. For the single arc prostate plans, no difference was observed between BDR and CVDR at the 3%/3 mm gamma level, although at the tighter tolerance of 2%/2 mm, the CVDR deliveries resulted in a higher pass rate. Similarly, for the complex two arc head‐and‐neck plans, the CVDR deliveries had a higher pass rate at all gamma levels for individual arcs. It is of interest to observe that the combined dose distributions (from both arcs) did not reflect this difference. Further analysis reveals that, generally, the Delta^4^ dose measurements of the first head‐and‐neck arc are systematically high, but are systematically low for the second arc (Fig. 6). As such, the combined dose distribution results in an acceptable gamma pass rate. The reason for this may be attributable to the way in which the dual arc plans are created. The SmartArc plans tended to produce one arc which conforms to the shape of the target volumes, and a second which is more heavily modulated to ensure a more uniform dose in the target, while maintaining the avoidance of organs at risk.

The study by Bertelsen et al.^(13)^ reported higher gamma pass rates for head‐and‐neck plans, which may be due to differences in treatment protocol, and choice of VMAT parameters (a single arc, 2° control point spacing, compared to a dual arc, 4° solution in this study). However, the results presented here are in agreement with those reported previously, in that they indicate a slight improvement in dosimetry with CVDR compared to BDR.

The fundamental difference between CVDR and BDR deliveries is the ability to select from a larger range of dose rates during treatment, which also allows higher gantry speeds to be selected. Using the EPID as an independent means of tracking MLC position, it was possible to determine the impact of these changes. While the mean MLC errors were small (head‐and‐neck plans gave 0.13 cm and 0.06 cm for CVDR and BDR, respectively), a statistically significant difference was observed between the delivery methods. This trend is similar to that noted by Bertelsen et al. However, the two methods are not directly comparable – the Bertelsen study uses the leaf error signal from the linac's service graphing function, whereas this study relates MLC position determined independently (from the EPID) with the planned position.

**Figure 7:**
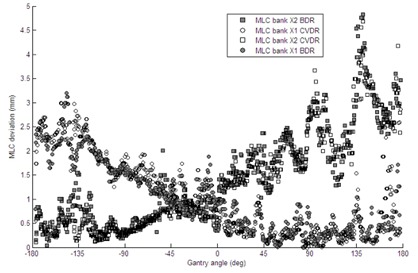
Scatter plot of mean MLC deviations from each leaf bank (X1 and X2) over all prostate patients, plotted against gantry angle. Initially, leaf bank X1 is traveling against gravity. Shaded boxes and circles indicate CVDR delivery.

While it has been observed that the faster CVDR deliveries result in a higher mean MLC deviation over each treatment, it is more difficult to determine the whereabouts of any systematic positioning errors during the arc. A plot of gantry angle versus MLC deviations (Fig. 7) indicates that the leaf bank which is traveling against gravity has larger deviations. However, this trend is observed to be similar for both BDR and CVDR deliveries. An investigation of MLC deviation against instantaneous gantry speed would be of interest, but this is a difficult parameter to determine independently during VMAT delivery. Future work may involve the use of an external inclinometer (such as that used with the Delta^4^ device) to reliably measure instantaneous gantry speed, and investigate any relationship to instantaneous MLC errors.

The dynamic monitoring of MLC position within the Elekta control system will temporarily interrupt the beam if a leaf error of < 0.4 cm is detected. With the move towards faster VMAT deliveries (through the use of CVDR, and potentially much higher dose rates^(24)^), the tolerance of this dynamic error monitoring may have to be tightened.

It is preferential for VMAT to be delivered with a dose rate which is closer to calibration and QA conditions.^(14)^ Utilizing the portal imager, it has been possible to confirm that CVDR delivery, with its higher mean dose rate, leads to a flatter and more stable beam over the duration of delivery. Figure 4 shows how the st. dev. of the beam profiles varies over the treatment. With the BDR system it is possible to observe ‘spikes’ in the beam flatness which occur when there are large changes in dose rate. The CVDR system does not appear to contain these spikes due to the smaller intervals between dose rates. While both delivery methods are dosimetrically robust, the CVDR system presents an advantage in terms of beam stability during delivery.

These results suggest that any negative dosimetric impact from MLC positioning which arises with the use of CVDR is smaller than the positive impact of the improved beam stability. It will be of future interest to determine what level of complexity can be achieved before the impact of MLC positioning errors becomes significant. It should also be noted that the ability of the linac to reach new aperture shapes is strongly dependant on the speed of the MLCs. In this study, the Elekta linac was fitted with standard 1 cm MLCi leaves. As VMAT becomes more widely used, modern MLC designs are placing greater importance on leaf speed, which will enable more complex changes in aperture shape without having to significantly reduce the dose rate or gantry speed.

It should be noted that the results presented here may be dependent on the planning system, and treatment protocols employed. At present this center uses VMAT for prostate treatments and selected head‐and‐neck sites. It will be of future interest to add to the small sample size considered in this study with more complex clinical sites, such as paraspinal tumours^(25)^ and medulloblastoma (whole central nervous system) treatments.^(26)^ Furthermore, it will be of use to investigate whether the EPID tracking and flatness measurements can be reproduced using other devices, such as a head‐mounted diode or ion chamber array.

## V. CONCLUSIONS

VMAT delivered with both continuously variable dose rate and binned dose rates provides high quality dosimetric verification for prostate and head‐and‐neck plans. The CVDR system, packaged with the Elekta Integrity software upgrade, is also capable of significantly shorter delivery times. Investigating two important components of the delivery, it was found that MLC positioning accuracy is slightly poorer with the faster CVDR deliveries, but that beam flatness and stability is improved compared to BDR. For complex VMAT deliveries, therefore, the superior beam stability (a result of the higher average dose rate with smaller intervals) appears to be the dominant factor in improved dosimetry for CVDR.

## ACKNOWLEDGMENTS

The authors would like to thank Elekta for its assistance with the installation of the Integrity control software, and David Ryder for his help with the preparation of this paper. The lead author's work is partly funded by an Elekta research grant.
